# Genomic and phenotypic biology of a novel *Dickeya zeae* WH1 isolated from rice in China: Insights into pathogenicity and virulence factors

**DOI:** 10.3389/fmicb.2022.997486

**Published:** 2022-10-28

**Authors:** Xiao-Juan Tan, Zhi-Wei Zhang, Jing-Jing Xiao, Wei Wang, Feng He, Xuan Gao, Bin Jiang, Liang Shen, Xu Wang, Yang Sun, Guo-Ping Zhu

**Affiliations:** ^1^College of Life Sciences, Anhui Provincial Key Laboratory of Molecular Enzymology and Mechanism of Major Diseases, Anhui Normal University, Wuhu, Anhui, China; ^2^Wuhu Qingyijiang Seed Industry Co., Ltd., Wuhu, Anhui, China

**Keywords:** *Dickeya zeae*, bacteria soft rot of rice, comparative genomics, pathogenicity determinants and virulence factors, phenotypic diversity

## Abstract

Soft rot caused by *Dickeya zeae* is an important bacterial disease affecting rice and other plants worldwide. In this study, Nanopore and Illumina sequencing platforms were used to sequence the high-quality complete genome of a novel *D. zeae* strain WH1 (size: 4.68 Mb; depth: 322.37x for Nanopore, 243.51x for Illumina; GC content: 53.59%), which was isolated from healthy rice root surface together with *Paenibacillus polymyxa*, a potential biocontrol bacterium against *D. zeae* strain WH1. However, the pure WH1 culture presented severe pathogenicity. Multilocus sequence analysis (MLSA) indicated that strains WH1, EC1, and EC2 isolated from rice were grouped into a clade differentiated from other *D. zeae* strains. The average nucleotide identity (ANI) and DNA-DNA hybridization (DDH) analyses demonstrated that WH1 was phylogenetically closest to EC2. Furthermore, the pathogenicity determinants and virulence factors of WH1 were mainly analyzed through genomic comparison with complete genomes of other *D. zeae* strains with high virulence (EC1, EC2, MS1, and MS2). The results revealed that plant cell wall-degrading extracellular enzymes (PCWDEs), flagellar and chemotaxis, and quorum sensing were highly conserved in all analyzed genomes, which were confirmed through phenotypic assays. Besides, WH1 harbored type I, II, III, and VI secretion systems (T1SS, T2SS, T3SS, and T6SS), but lost T4SS and T5SS. Like strains MS1 and MS2 isolated from bananas, WH1 harbored genes encoding both capsule polysaccharide (CPS) and exopolysaccharide (EPS) biosynthesis. The results of pathogenicity assays demonstrated that WH1 produced severe soft rot symptoms on potato tubers, carrots, radishes, and Chinese cabbage. Meanwhile, WH1 also produced phytotoxin(s) to inhibit rice seed germination with an 87% inhibitory rate in laboratory conditions. More importantly, we confirmed that phytotoxin(s) produced by WH1 are different from zeamines produced by EC1. Comparative genomics analyses and phenotypic and pathogenicity assays suggested that WH1 likely evolved through a pathway different from the other *D. zeae* strains from rice, producing a new type of rice foot rot pathogen. These findings highlight the emergence of a new type of *D. zeae* strain with high virulence, causing soft rot in rice and other plants.

## Introduction

*Dickeya* spp. are Gram-negative and causative agents of a wide range of bacterial soft rot and have been listed among the top 10 most important bacterial phytopathogens due to the high costs and economic consequences they incur (Mansfield et al., 2012). Currently, the *Dickeya* genus encompasses 12 recognized species (Boluk et al., 2021). Of which, *D. zeae* strains were isolated from a wide and diverse range of hosts, causing serious economic losses in crop yield in different parts of the world, especially for rice, potato, pineapple, and banana (Hu et al., 2018; Feng et al., 2019; Boluk et al., 2021). Infected rice plants by *D. zeae* have a dark brown decay of the tillers at the site of infection with a bad smell, which later leads to the collapse of the entire plant (Hussain et al., 2008). In addition, *D. zeae* strains can reside on the surface and/or within the intercellular spaces of plant organs without exhibiting symptoms (Pérombelon, 1992). The reason these bacteria can grow on some plants without symptoms but cause devastating diseases in others remains unclear (De Boer, 2002; Van Gijsegem et al., 2021). However, when in a susceptible host, these strains may persist for months until the environmental conditions are not favorable to disease initiation (De Boer, 2002; Kraepiel and Barny, 2016). Additionally, they can persist overwinter in contaminated plant residue and soil. Under certain conditions, such as decreasing O_2_ concentration, high temperature, high humidity, and there being water film on the surface of the plant organs, latency is broken and the bacteria start to grow and produce plant cell wall degrading enzymes (PCWDEs) leading to disease development in susceptible hosts (Bez et al., 2021).

Generally, *D. zeae* strains infect plants through three main steps: (1) adhesion to plant surface and penetration in the plant tissues, either via wound sites or through natural openings such as stomata; (2) invasion of apoplasts; and (3) plant cell wall degradation (Reverchon and Nasser, 2013). Some typical virulence factors are involved in these processes, such as type I–VI secretion systems (T1SS–T6SS), PCWDEs, flagella, and flagella-mediated motility, quorum sensing signal molecules acylhomoserine lactone (AHL) and Vfm, and biofilms (Zhang et al., 2022). A previous study showed that the zeamines produced by *D. zeae* EC1 were important virulence factors for rice, but antagonists for many bacteria and fungi (Liao et al., 2014). However, the *zms* gene cluster encoding zeamines biosynthesis was absent in the genome of *D. zeae* strain EC2 (Zhang et al., 2020). Additionally, other virulence determinants are present among *D. zeae* strains, such as T3SS, which is present in strain EC1 but absent in strain EC2 (Zhou et al., 2015; Zhang et al., 2020). Therefore, the diversity involved in virulence factors would be present in the genomes of *D. zeae* strains, even though they were isolated from the same host.

In this study we obtained a complete genome sequence of *D. zeae* strain WH1, isolated from the rice root surface, using Illumina next-generation sequencing and Nanopore sequencing technologies. The genome sequence was annotated and compared with the representative genome sequences of other *D. zeae* strains (EC1, EC2, MS1, and MS2), focusing on virulence determinants and potential regulatory mechanisms. Substantial phenotype and pathogenicity assays were performed for strain WH1. The genome-wide comparison and phenotypic and pathogenicity assays could help to clarify the differentiation among *D. zeae* strains from rice and their genetic adaptions to the host. This information directs attention to this newly emerging rice pathogen to provide better insights into the early prevention of rice foot rot disease.

## Materials and methods

### Strain WH1 isolation and characterization

Three healthy rice roots were collected from the field of Nanling, Wuhu City, Anhui Province, China, in August 2021. The rice roots with few adherent soils were cut into pieces and placed into 10 mL of sterile water. The mixtures were incubated at 25°C for 30 min with shaking at 150 rpm. The mixture at different dilutions with sterile water was spread onto LB agar plates and incubated at 25°C for 48 h. Single colonies were picked and sub-cultured three times. Pure cultures were used to screen antagonists against rice pathogenic fungi. One strain, WH1, had the strong ability to inhibit the growth of pathogenic fungi (data not shown). The strain was identified as *Dickeya* genus through sequencing of the 16S rRNA gene using 27F/1492R primers. Subsequently, the strain was further confirmed through Koch’s postulates. Additionally, the screening of antagonistic bacteria against WH1 was performed according to the methods previously described (Li et al., 2020). The screened antagonistic bacteria were identified through sequencing of the 16S rRNA gene. All isolated bacteria were stored in glycerol at –80°C at Anhui Normal University, China. The strain WH1 was mainly investigated in this study. For subculture, the strain was grown at 28°C overnight in LB broth (10 g/L tryptone, 5 g/L yeast extract, and 10 g/L NaCl, pH7.0) in an incubator shaker at 150 rpm.

### Genomic DNA extraction and genome sequencing

The strain WH1 was incubated to logarithmic phase at 28°C and its genomic DNA was extracted using the TIANamp Bacterial DNA kit (Tiangen Biotech, Beijing, China) according to the manufacturer’s instructions. The quality of the obtained genomic DNA was detected with agarose gel electrophoresis, NanoDrop One Spectrophotometer (NanoDrop Technologies, Wilmington, DE, USA), and Qubit 3.0 Fluorimeter (Life Technologies, Carlsbad, CA, USA), respectively. The DNA libraries for MinION Nanopore sequencing and Illumina sequencing were constructed, respectively. DNA sequencing was respectively carried out on the PromethION sequencer (Oxford Nanopore Technologies, Oxford, United Kingdom) and NovaSeq 6000 (Illumina, USA) platforms at BENAGEN Technologies, Wuhan, China.

### Genome assembly and annotation

Unicycler (version 0.5.0) software was used to assemble short reads from the Illumina platform and long reads from the MinION Nanopore platform (Wick et al., 2017). Gene prediction was performed using Prokka (version 1.14.6) software (Seemann, 2014); Aragorn was used to predict the tRNA of the genome (Laslett and Canback, 2004); rRNAmmer was used to predict genomic rRNA sequences (Lagesen et al., 2007). Gene function annotation was performed using the local BLAST method to compare the predicted gene sequences with GO (Ashburner et al., 2000), KEGG (Kanehisa et al., 2004), COG (Tatusov et al., 2000), UniProt (Apweiler et al., 2004), and NR databases, respectively, to obtain the corresponding annotation information. The genomic sequence was submitted to the NCBI GenBank Genome database under the accession number CP101400.

### Phylogenetic analysis of *Dickeya* strains

Sixteen *Dickeya* strains with complete genome sequences were used to extract the gene sequences of *apt*D, *gyr*B, *inf*B, and *rpo*B for multilocus sequence analysis (MLSA). The concatenated sequences of these four genes were processed on MEGA5.2 using a neighbor-joining algorithm with 1,000 bootstrapped replications (Tamura et al., 2011). To obtain an estimate of overall genomic similarities, a pairwise comparison of the WH1 genome with 15 genomes of other *Dickeya* strains was performed using the average nucleotide identity (ANI) based on the nucleotide MUMmmer algorithm (ANIm) in the Jspecies Web server (Richter et al., 2016). In addition, *in silico* DNA-DNA hybridization (isDDH) values were calculated using the Genome-to-Genome Distance Calculator (GGDC) with formula 2 and BLAST + alignment.^1^ The cut-off values of 96 and 70% were assigned as a species-delineation framework for ANI and isDDH, respectively (Goris et al., 2007).

### Genomic comparison between WH1 and other *Dickeya zeae* strains

The complete genome of WH1 was compared with the other four complete genomes of *D. zeae* strains (EC1, EC2, MS1, and MS2). Their genome sequences were retrieved from the NCBI genome database. The basic genomic profile features of these four strains were taken from the NCBI GenBank database and the bioinformatics resource center proteome comparison tool of the pathosystems resource integration center (PATRIC) Web Server (Davis et al., 2020). To identify the lists of shared and unique gene clusters, Pan-genome analysis was performed using Pan-Genomes Analysis Web Server (Chen et al., 2018). The orthologous gene clusters were identified by the Gene Family (GF) method set to its default parameters.

The soft rot bacteria within *Dickeya* genus macerate the plant tissue and acquire nutrients from the dead cells. Most pathogenicity determinants related encoding genes, including PCWDEs, type secretion systems (types I–VI), extracellular polysaccharides (such as CPS and EPS), bacterial attachment operons (type IV pili), flagella and chemotaxis, zeamine synthesis, quorum sensing systems, and biofilm formation, have been described in strains EC1, EC2, MS1, or MS2 (Feng et al., 2019; Zhang et al., 2020, 2022; Liu et al., 2022). In this study, we mainly analyzed and compared the similarities, differences, or absence of gene encoding virulence determinants between WH1 and these four *D. zeae* strains.

### Extracellular enzyme activity assays

Pectate lyases (Pel), cellulases (Cel), and proteases (Prt) produced by *Dickeya* genus were the major causes resulting in foot rot in rice and other plants (Boluk et al., 2021). Therefore, their activities were detected according to the protocols as previously described with a few modifications (Chatterjee et al., 1995; Hu et al., 2022). In brief, the media for enzyme activity assays as follows: (1) Pel assay medium (per liter): 10 g polygalacturonic acid, 10 g yeast extract, 0.38 μmol CaCl_2_, 100 mmol Tris-HCl, 15 g agar, pH8.5; (2) Cel assay medium (per liter): 1.0 g carboxymethyl cellulose, 3.8 g Na_3_PO_4_, 15 g agar, pH7.0; (3) Prt assay medium: LB agar medium containing 1% (v/v) skimmed milk. The media were poured and allowed to solidify and 5 mm diameter wells were punched into the agar and sealed the bottom with worm 1.5% agar solution. 25 μL overnight cultures were applied to each well, and plates were incubated at 28°C. After 15 h, Pel assay plates were flooded with 1 M HCl; Cel assay plates were flooded with 1% Congo red solution for 1 h and washed twice with 1 M NaCl solution. Next, the treated Pel and Cel assay plates were incubated at room temperature for 24 h and the diameter of haloes around the wells were measured. For Prt assay, the haloes around the wells were measured after 24 h without any treatment. Each treatment was carried out at least three times, and all assays were repeated at least three times.

### Flagellum-mediated motility assays

These assays were performed according to the methods as previously described (Boluk et al., 2021). Media for swimming (per liter contains 10 g tryptone, 5 g NaCl, and 3 g agar) and swarming (per liter contains 5 g peptone, 3 g yeast extract, and 4 g agar) assays were prepared. One microliter of overnight bacterial culture was spotted onto the center of a plate (60 mm diameter) containing about 10 mL of each medium. The plates were incubated at 28°C for 20 h before measurement of the diameters of each bacterial motility zone. Each experiment was repeated at least three times in triplicate.

### Assays of exopolysaccharide production

Exopolysaccharide (EPS) was detected in WH1 culture according to a previous study with minor modifications (Hu et al., 2018). In brief, a single colony of WH1 strain was picked and transferred into a 2 mL LB medium and incubated at 28°C overnight. Afterward, 1 mL of which was applied into 100 mL LB medium and grown with shaking at 150 rpm for 12 h. Cultures were centrifuged at 8,000 rpm for 10 min, and then at 4,000 rpm for 20 min to obtain 80 mL supernatants. Double volumes of ethanol were added to the supernatants, mixed thoroughly, stored at 4°C overnight for precipitation, and subjected to centrifuge at 8,000 rpm for 20 min. Finally, supernatants were discarded and pellets were weighed after drying at 55°C overnight. The experiment was repeated three times in triplicate.

### Biofilm formation assays

An overnight bacterial culture was diluted at 1:100 with LB medium; 200 μL of the culture was added into each well of 96-well microtiter plates and statically incubated at 28°C for 20 h. For qualification of the total biofilm mass, the suspension culture was removed and the plate was washed three times with PBS. After drying for 2 h at 60°C, the biofilm was stained with 50 μL of 0.1% crystal violet (w/v) for 15 min. The wells were also washed three times with PBS to remove unbound crystal violet dye and dried for 2 h at 60°C. Biofilms formed in 96-well plate were observed. Additionally, the biofilms developed in a 48-well microtiter plate containing one glass coverslip per well were used during observation with a fluorescent microscope, as previously described (Merritt et al., 2005). The plate was statically incubated at 28°C for 20 h. The AO/EB staining kit (Sango Biotech, Shanghai, China) was used to stain biofilms developed on glass coverslip after being washed three times with PBS to discard planktonic cells. The images of biofilms were observed through the fluorescent microscope (Leica Microsystem, Germany).

### Acylated homoserine lactones production assay

*Chromobacterium violaceum* CV026 is usually used as a biosensor to visualize acylated homoserine lactones (AHLs) with N-acyl side chains from C4 to C8 in length production by Gram-negative bacteria (Pu et al., 2022). In this study, one colony of CV026 was inoculated into 2 mL of LB medium and incubated at 28°C overnight. The CV026 cultures were added to a warm LB agar (1.5%) medium at a ratio of 1:100, and then the mixtures were poured immediately over the surface of LB agar plates prepared in Petri dishes. When the overlaid agar solidified, a well (5 mm diameter) was made in the center of each plate and sealed the bottom with warm agar solution, 25 μL of overnight WH1 culture (10^9^ CFU/mL) was added to the well. Meanwhile, 25 μL of overnight *Pseudomonas aeruginosa* PAO1 culture (10^9^ CFU/mL) was performed under the same conditions as the reference. Violacein halo production was observed after being incubated at 28°C for 24 h. The diameter of the violacein halo was also measured.

AHL production was determined by inoculating 200 μL of an overnight culture of WH1 into 200 mL of LB. After 20 h incubation at 28°C, the sterile supernatant was polled and extracted with acidified ethyl acetate according to a protocol described previously (Zhou et al., 2017). The AHL produced by WH1 was analyzed using an HPLC system (Shimadzu, Japan) equipped with a C18 column by UV absorbance at 210 nm. The mobile phase A was water, and the mobile phase B was methanol. The following rate was set as 0.8 mL/min. The injection volume was 20 μL. The peak corresponding to 3OC6-HSL was identified according to the retention time of commercial 3OC6-HSL standard (Aladdin, Shanghai, China) with the same HPLC protocol.

### Pathogenicity assays

Thirty rice seeds were put into 10 mL of sterile water containing 1 mL of WH1 overnight culture and incubated at room temperature for 5 h. The rice seeds were rinsed three times with sterile water and transferred onto the top of two moistened filter papers placed on a sterilized plate at room temperature. Rice seeds were treated with the same volume of sterile water as a negative control. The rice seeds were then incubated at 33°C under 8 h dark/16 h light with supplementation of sterile water when necessary. The seed germination rate was measured after 7 days.

To investigate the pathogenicity of strain WH1 on other plants, we selected potato tubers, carrots, radishes, and Chinese cabbage. The plants were washed with deionized water and then surface-disinfected with 70% ethanol, cut into slices, and dried for 30 min in a bio-safety hood. 25 μL of overnight culture was inoculated on the center of the tissue slices. After inoculation, tissue slices were placed on wet sterilized filter papers in a sterile double-layer drain basket and kept in a growth chamber in conditions of 28°C and 75% relative humidity. The lesions were measured every day for 3 days. Tissue slices inoculated with LB medium were used as a control.

### Zeamine detection assay

The zeamine detection assay was performed according to a method previously described with a few modifications (Chen et al., 2016). In brief, plates were prepared by pouring about 10 mL of LB agar medium, overlaid with 5 mL of 1% agar containing 50 μL overnight culture of fresh *Escherichia coli* DH5α after solidification. 25 μL of overnight WH1 culture in LS5 medium (per liter contains: 5.25 g K_2_HPO_4_, 2.25 g KH_2_PO_4_, 10.0 g sucrose, 3.6 g NH_4_NO_3_, 1.0 g KCl, 0.25 g MgSO_4_, pH7.0) were added to the wells in plates. The bioassay plates were incubated at 28°C for 24 h before photography. The diameter of the inhibition zone was measured.

### Statistical analysis

All assays were carried out at least three times independently unless otherwise stated. The results obtained were summarized in figures as mean ± standard deviations. Statistical significance was evaluated using a two-tailed Student’s *t* test. A *p*-value < 0.05 was considered significant.

## Results

### WH1 isolated from rice root was classified as *Dickeya zeae* based on phylogenetic analysis

**FIGURE 1:**
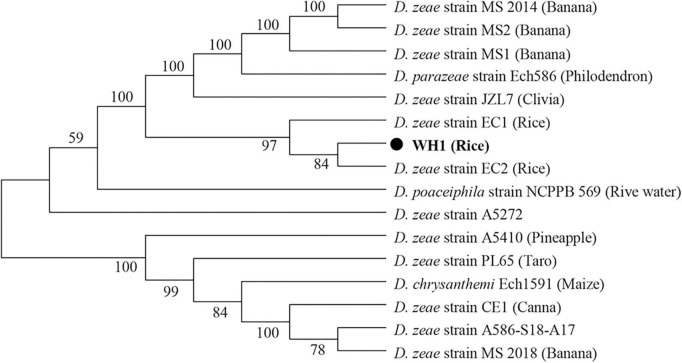
Phylogenetic placement of *Dickeya zeae* strain WH1. Phylogenetic analysis of *Dickeya s*trains based on concatenated sequences of housekeeping genes, *apt*D, *gyr*B, *inf*B, and *rpo*B, 16 Dickeya strains with complete genomes were included in a phylogenetic analysis with a neighbor joining algorithm and bootstrapped at 1,000 replications.

In this study, although the strain WH1 was isolated from healthy rice root in Wuhu City, China (Eastern China), the pure culture was inoculated to the rice root, and typical soft rot symptoms were observed (data not shown), validating its role as rice pathogen. Meanwhile, we isolated one strain, *Paenibacillus polymyxa*, from the healthy rice root, which can inhibit the growth of strain WH1 (Supplementary Figure 1). Therefore, it is not surprising that WH1 was isolated from healthy rice roots. Additionally, we found that WH1 can inhibit the growth of *Candida albicans*, while *P. aeruginosa* PAO1 can inhibit the growth of WH1 (Supplementary Figure 2). To identify the taxonomic status of strain WH1, MLSA was performed based on the complete sequences of four housekeeping genes including *apt*D, *gyr*B, *inf*B, and *rpo*B. Phylogenetic analysis of concatenated sequences of four genes distinctly separated *D. zeae* strains from other species. Among these *D. zeae* strains, EC1, EC2, and WH1 isolated from rice were grouped into a clade differentiated from other *D. zeae* strains. WH1 was placed at the same branch with EC2 isolated from rice (Figure 1). ANI analysis indicated that the ANI values between WH1 and previously identified strains from rice ranged from 97.34 to 98.28% and were higher than the cut-off value (96%) for species delineation. Meanwhile, the isDDH values between WH1 and *D. zeae* strains isolated from rice ranged from 76.3 to 83.9%, which were also higher than the cut-off value (70%) (Table 1). In summary, these data establish that strain WH1 belongs to the species *D. zeae*.

**TABLE 1:** Average nucleotide identity (ANI) and *in silico* DNA–DNA hybridization (isDDH) values between the genomes of *Dickeya zeae* strain WH1 and other characterized *Dickeya* species.

*Dickeya* strains	Host/Source	ANI value (%)	isDDH value (formula 2) (%)
***Dickeya zeae* strain EC2**	Rice	98.28	83.9
*Dickeya zeae* strain A5272	–	97.36	76.6
*Dickeya zeae* strain EC1	Rice	97.34	76.3
*Dickeya zeae* strain A5410	Pineapple	96.41	69.4
*Dickeya zeae* strain MS1	Banana	95.17	60.8
*Dickeya zeae* strain MS2	Banana	95.06	60.1
*Dickeya zeae* strain MS 2014	Banana	95.06	60.1
*Dickeya zeae* strain MS 2018	Banana	95	59.8
*Dickeya zeae* strain PL65	Taro	94.59	57.7
*Dickeya zeae* strain JZL7	Clivia	94.58	57.6
*Dickeya zeae* strain CE1	Canna	94.55	57.5
*Dickeya zeae* strain A586-S18-A17	-	94.52	57.3
*Dickeya zeae* strain Ech586	Philodendron	94.49	57
*Dickeya chrysanthemi* strain Ech1591	Maize	87.28	30.4
*Dickeya poaceiphila* strain NCPPB 569	River water	87.2	30

### Genomic differences between WH1 and other *Dickeya zeae* strains

**FIGURE 2:**
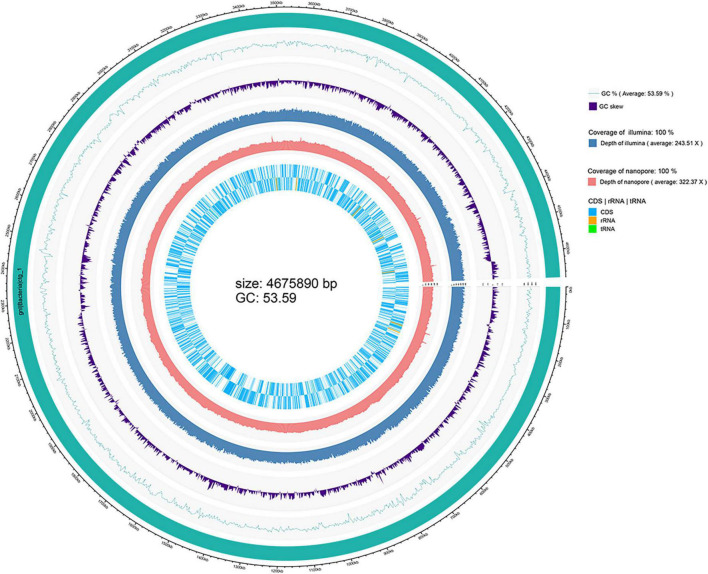
Circular visualization of the complete genome of *Dickeya zeae* strain WH1.

The depth of the assemblies was 243.51 and 322.37 for Illumina and Nanopore platforms, respectively. The complete genome of strain WH1 consisted of a single circular chromosome of 4,675,890 base pairs with a 53.59% GC content. General features of the genome are listed in Table 2, and a detailed circular map is shown in Figure 2. In total, 22 predicted rRNA genes and 76 tRNA genes were identified, and 86.92% of nucleotides are predicted to coding proteins. By a combination of coding protein prediction and homology search, 4096 coding DNA sequences (CDSs) with an average length of 979 bp were identified on the genome. Of these, 274 CDSs were annotated by COG function groups and classified into 26 COG categories. Meanwhile, 1,572 CDSs were annotated by KEGG. 5.81% (238) of total ORFs were annotated as hypothetical proteins. Moreover, four representative *D. zeae* strains, EC1, EC2, MS1, and MS2, were isolated from rice and banana, resulting in severe soft rot disease. Their genome sequences were downloaded from NCBI and used for genomic comparison analysis. Their genome features are also listed in Table 2.

**TABLE 2:** General genomic information for five *Dickeya zeae* strains isolated from rice and banana.

Genome features	WH1	EC1	EC2	MS1	MS2
Year of isolation	2021	–	2016	2009	2014
Location	Nanling, Wuhu, China	Guangzhou, China	Qingyuan, Guangzhou, China	Nansha, Guangzhou, China	Nansha, Guangzhou, China
Length (bp)	4,675,890	4,532,364	4,575,125	4831702	4,740,052
% GC	53.59	53.43	53.34	53.3	53.44
CDS	4096	3826	3880	4130	4076
rRNAs	22	22	22	22	22
tRNAs	76	88	75	75	75
ncRNAs	147	12	9	10	6
Pseudogenes	297	65	75	88	37
Hypothetical proteins	238	234	224	301	248
Virulence factor*	71	71	72	70	71
Transporter*	185	176	185	183	186
Antibiotic resistance*	40	49	43	54	54
Drug target [TTD]*	27	27	27	26	27

GC, guanine-cytosine; CDS, coding sequence regions; TTD, Therapeutic target database.

*Data retrieved from the bioinformatics PATRIC webserver.

**FIGURE 3:**
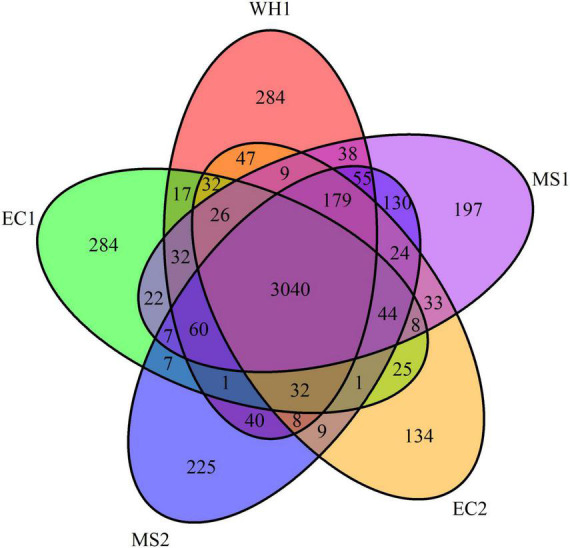
Venn diagram representing the numbers of common or specific gene clusters among analyzed *Dickeya zeae* strains in PGAP analysis.

Pan-genomes analysis was used to develop a robust comparative genomics analysis. The results showed that the number of core gene clusters among the five genomes was 3,040. The number of specific clusters in WH1, EC1, EC2, MS1, and MS2 were 284, 284, 134, 197, and 225, respectively (Figure 3). The unique clusters in WH1 contained some important encoding genes, such as transporter proteins, transposases, endonucleases, and toxin-antitoxin systems. Besides, the unique clusters contained 113 genes encoding hypothetical proteins (Supplementary Table 1). However, the unique clusters in EC1 contained genes encoding zeamine biosynthesis (Supplementary Table 2).

### Plant cell wall-degrading extracellular enzymes

Most of these extracellular enzymes, pectate lyases (Pel), and other pectinase such as pectin methylestrases (Pem) and pectin lyase (Pnl) produced by *D. zeae* strains have been shown to play a major role in the virulence and tissue maceration (Boluk et al., 2021). The genome WH1 contained a total of 14 genes encoding pectin degradation enzymes (Supplementary Table 3). These pectin degradation genes were highly conserved in various *D. zeae* strains, except that *pel*B encoding pectate disaccharide lyase was absent in the MS1 strain and *pem*B encoding pectinesterase B precursor was absent in all analyzed strains (Supplementary Table 3). Although the *peh*KNVWX genes encoding polygalacturonases were also conserved in *D. zeae* strains, parts of which (*peh*NVW) were absent in all analyzed genomes. In addition, the endoglucanase genes (*cel*Y and *cel*H) and beta-glucosides encoding genes (*bgl*ABCD and *bgx*A) are involved in the degradation of cellulose to glucose. Although these genes were highly conserved in the *Dickeya* genus, *bgl*C was absent in strains MS1 and MS2 isolated from banana, *bgl*D was just present in EC1, *cel*Y was just present in WH1 and EC1. In addition, the homologies of *bgl*B and *bgl*C in WH1 were 53 and 52% with EC1, respectively, although both were isolated from rice. The xylanases (*xyl*BAFGHR and *xyn*A) genes are involved in the degradation of xylan and xyloglucan mainly present in plant cell walls (Boluk et al., 2021). All genomes of analyzed *D. zeae* strains contained these genes. In summary, these *pel*, *peh*, and *cel* genes were conserved in genomes of *D. zeae* strains (Supplementary Table 3).

The *prt*ABCDEFG gene cluster encoding four proteases and three protease secretion-associated proteins (T1SS) was located on the positive strand of WH1, EC1, MS1, and MS2 chromosomes, but located on the negative strand of the EC2 chromosome. Alignment with other *D. zeae* strains showed that they were highly conserved in the genome of WH1 (Supplementary Table 3).

### Secretion systems

The type II secretion system (T2SS) is used by gram-negative bacteria to translocate extracellular proteins across the outer membrane (Tan et al., 2021). In *D. zeae* strains, the T2SS is encoded by the *out* genes to allow the secretion of several proteins including most pectinases. Therefore, T2SS plays an important role in the pathogenicity of *Dickeya* spp. on host plants. WH1 chromosome harbored a T2SS gene cluster (*out*OMLKJIHGFEDCBS), covering about 13 kb with 14 ORFs. The gene cluster shared high similarity with that of other analyzed *D. zeae* strains (similarity 99%) at the amino acid level (Supplementary Table 4). Therefore, the *out* gene cluster was highly conserved among *D. zeae* strains.

Besides T2SS plays an important role in the pathogenicity of *Dickeya* spp., T3SS was also confirmed as a pathogenicity factor in many phytopathogenic bacteria (Hu et al., 2022). Similarly, the *hrp* genes in *Dickeya* spp. have also been reported to play an important role in their pathogenesis and interaction with host plants (Hu et al., 2018). In the genome of WH1, a large *hrp* gene cluster spanning 27.3 kb was identified, which was composed of 29 genes, while 27 genes in EC1, three genes in EC2, 26 genes in MS1, and 31 genes in MS2 (Supplementary Table 4). The homologies of *hrp* gene cluster in WH1 were high with EC1, MS1, and MS2, arranged from 80 to 100% at the amino acid level.

The Type IV secretion system constitutes *vir*B1-11 genes and functions in conjugation, pathogenicity, and DNA release/uptake (Zhou et al., 2015). Some *D. zeae* strains, such as EC1, Ech586, MS1, and A5410, harbored *vir*B-T4SS cluster, while the cluster was not found in WH1 and EC2, just *vir*B1 and *vir*B2 were present in MS2 (Supplementary Table 4).

The type VI secretion system (T6SS) was identified to take part in bacterial pathogenicity and host colonization (Chen et al., 2015; Lin et al., 2017). Many studies showed that T6SS functions in various biological processes, such as mediating cooperative or competitive interactions between bacteria and eukaryotes, and bacterial biofilm formation (Chen et al., 2015). T6SS is typically encoded by a cluster of 12–20 genes, with a minimum of 13 genes for the production of a functional apparatus (Zhou et al., 2015). In this study, a gene cluster encoding T6SS was present in *D. zeae* WH1, spanning 44 kb with 37 ORFs. In this gene cluster, in addition to 17 genes described previously with high identity at the amino acid level, there are 20 additional ORFs inserted between *vgr*G and *tss*B genes in genome WH1, while 16 genes in EC1, 22 genes in EC2, 21 genes in MS1, and 20 genes in MS2. For these inserted genes, two genes encoding ankyrin repeat proteins were present in WH1 and EC1, while three genes were in EC2, MS1, and MS2. Additionally, two genes encoding hypothetical proteins were inserted between both genes encoding ankyrin repeat proteins in WH1. IS family transposase was present in EC2 and MS1, but absent in WH1, EC1, and MS2. Two genes encoding plasmid stabilization system proteins ParDE were just found in WH1 and EC1 (Supplementary Table 4). In summary, different genes were inserted between *vgr*G and *tss*B of the T6SS gene cluster in all analyzed genomes.

### Flagellar and chemotaxis genes

Motility and chemotaxis are essential for phytopathogens when searching for favorable sites to enter into the plant apoplast. Therefore, they play important roles in plant infection by *Dickeya* spp. The flagellar biosynthesis and chemotaxis clusters constitute 20 *fli* genes, 14 *flg* genes, and 5 *flh* genes, involved in the flagella synthesis, four flagellar rotation genes and six chemotaxis associated genes were present and highly conserved in analyzed *D. zeae* genomes. Interestingly, two *fli*C encoding flagellin FliC were found in EC1, MS1, and MS2 genomes, but absent in WH1 and EC2 genomes. Moreover, there were 7 additional genes inserted between *fli*A and *fli*C genes in the WH1 genome, encoding a class I SAM-dependent methyltransferase (ctg_02571), a glycosylase (ctg_02573), two hypothetical proteins (ctg_02572 and ctg_02575), a WbqC family protein (ctg_02574), a DegT/DnrJ/EryC1/StrS family aminotransferase (ctg_02576), and a methyltransferase regulatory domain-containing protein (ctg_02577), which were not found in other four *D. zeae* genomes (Supplementary Table 5). The type IV pilus biogenesis encoding system consists of *pil*W, *pil*T, *pil*ABC, and *pil*MNOPQ genes. The *pil*W and *pil*T genes were located distant from the type IV pilus biogenesis cluster. They were highly conserved in *D. zeae* genomes (Supplementary Table 5).

### Polysaccharides biosynthesis genes

Besides lipopolysaccharide (LPS) as a component of the cell wall in Gram-negative strains, *Dickeya* genus can biosynthesize other polysaccharides, such as capsular polysaccharide (CPS) and exopolysaccharide (EPS). CPS can form a discrete capsule around the cell and is intimately associated with the cell surface; EPS is one of the important virulence factors for bacterial phytopathogens and the main toxic factors leading to water-logging and wilting on plants after infection (Hu et al., 2018). Both are important factors in infection and biofilm formation. In our analysis, both clusters were present in WH1, MS1, and MS2 genomes, but the CPS cluster was absent in EC1, and the EPS cluster was absent in EC2 (Supplementary Table 6).

### Quorum sensing systems

Many Gram-negative bacterial pathogens utilize the *lux*I/*lux*R quorum sensing system to regulate the expression of virulence genes and biofilm formation (de Kievit, 2009). Generally, *lux*I encodes a synthase for the production of AHL family quorum sensing signals, and *lux*R encodes an AHL signal receptor. Upon interaction with the AHL signal, LuxR becomes an active transcription factor and hence modulating the expression of virulence genes. Previous studies showed that N-3-oxohexanoyl-homoserine lactone (3OC6-HSL), *N*-3-oxo-octanoyl-homoserine lactone (3OC8-HSL), *N*-hexanoyl-homoserine lactone (C6-HSL) and *N*-decanoyl-homoserine lactone (C10-HSL) were produced by *Dickeya* genus (Liu et al., 2022). Of which, 3OC6-HSL was produced by *D. zeae* EC1 encoding by the *lux*I homologous *exp*I (Zhang et al., 2021). A Blast search of the WH1 genome revealed only one copy of *exp*I and one well-conserved *lux*R homolog *exp*R. ExpI produced by WH1 was highly homologous with the other four *D. zeae* strains (>99%) at the amino acid level and these four strains produce 3OC6-HSL (Supplementary Table 7). Therefore, WH1 would produce 3OC6-HSL, which was confirmed using HPLC (Data shown below description).

Interestingly, a novel QS system *vfm* gene cluster upstream of the *exp*I/*exp*R was found in the WH1 and other genomes analyzed in this study (63–100% identity at amino acid level). The gene cluster was originally identified in *D. dadantii* 3937 isolated from the lesion spot of a wilted African violet and was associated with the regulation of virulence factor production and pathogenesis (Potrykus et al., 2018). The *vfm* gene cluster, WH1, EC1, EC2, and MS2 genomes contained 26 genes including *vfm*AZBCDEFGHIJXWVUTSRQPOMNLKY, while the MS1 genome contained 27 genes with one gene encoding fatty acid CoA ligase family protein inserted between *vfm*N and *vfm*M (Supplementary Table 7). However, the biological significance of various QS systems in WH1 and other *Dickeya* species needs to be further investigated.

### WH1 produces toxins different from zeamines produced by EC1

*Dickeya zeae* EC1 produces at least two polyamino phytotoxins and antibiotics, zeamines, which were shown to be the major virulence determinants, affecting rice seed germination and inhibiting the growth of *E. coli* DH5α (Zhou et al., 2015; Lv et al., 2022). However, no zeamine cluster (*zms*O-*zms*N) was found in the WH1 and EC2 strains from rice and MS1 and MS2 strains from banana (Supplementary Table 8). Although zeamine biosynthesis genes were absent in the WH1 genome, the strain still inhibited rice seed germination, and the ability to infect other plants was not reduced (data shown in results of pathogenicity assays). Therefore, the above analysis indicated that the zeamines might not be a necessary virulence factor for this new strain from rice. Other virulence factors could be present in strain WH1 and need to be further investigated.

### Phenotype assays

**FIGURE 4:**
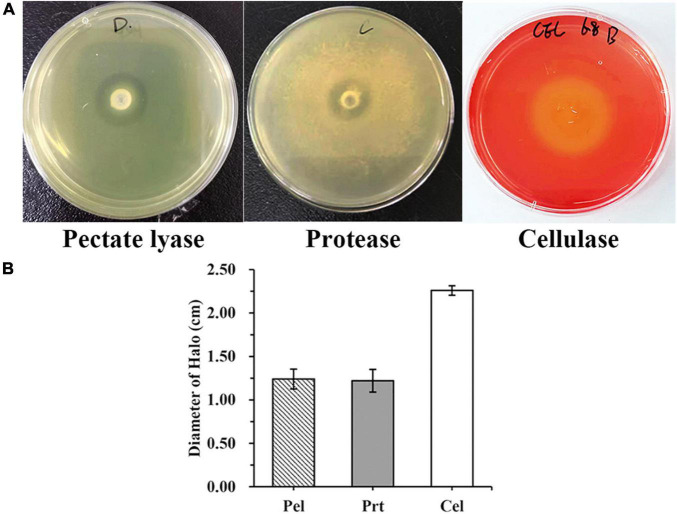
Extracellular cell wall-degrading enzymes produced by *Dickeya zeae* strain WH1. **(A)** Pectate lyase (Pel), Protease (Prt), and Cellulase (Cel) plates; **(B)** indicates the production of Pel, Prt, and Cel from *D. zeae* strain WH1.

**FIGURE 5:**
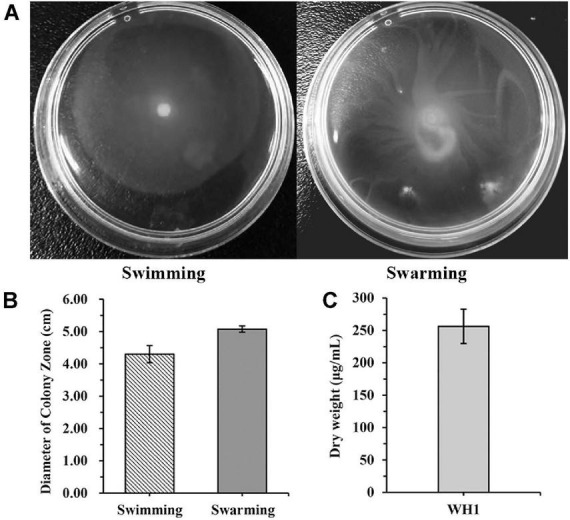
Swimming and swarming motilities and exopolysaccharide production of *Dickeya zeae* strain WH1. **(A)** Swimming and swarming capacities were observed in a semi-solid medium after 20 h at 28°C, respectively; **(B)** quantification of swimming and swarming abilities; **(C)** dry weight of EPS produced by *D. zeae* strain WH1.

**FIGURE 6:**
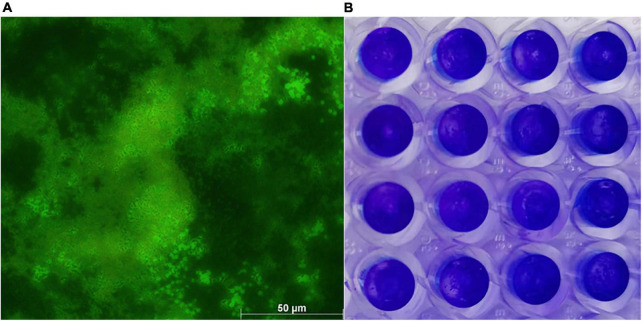
*Dickeya zeae* strain WH1 biofilms. **(A)** Images are presentative of *D. zeae* strain WH1 biofilm grown on a glass coverslip in a 48-well plate for 20 h, scale bar 50 μm; **(B)** biofilm developed in a 96-well plate for 20 h was stained with 0.1% crystal violet.

WH1 genome harbored the genes encoding proteases (*prt* cluster), pectate lyases (*pel* cluster), and cellulases (*cel*5Z, *cel*H, and *cel*Y). The strain produced Prts, Pels, and Cels in the plate assays (Figure 4). Moreover, WH1 generated large swarming (diameter greater than 50 mm) and swimming (diameter greater than 40 mm) diameters when incubated at 28°C for 20 h (Figures 5A,B). Both CPS and EPS clusters were found in the WH1 genome, which plays important roles in biofilm formation and pathogenicity for *D. zeae* strains. The results showed that WH1 has the ability to produce exopolysaccharides (Figure 5C). In addition, WH1 produced thick biofilm in a 96-well plate and the biofilms were verified with fluorescent images (Figure 6).

**FIGURE 7:**
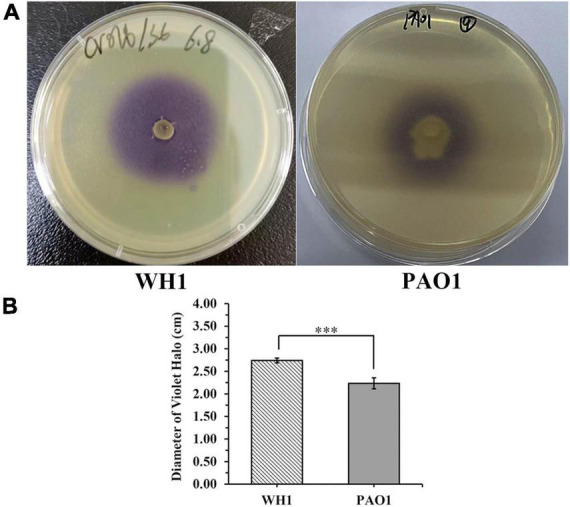
Acylhomoserine lactones (AHLs) production by *Dickeya zeae* strain WH1 and *Pseudomonas aeruginosa* PAO1 at the same conditions. **(A)** Images are presentative of WH1 and PAO1 grown on *Chromobacterium violaceum* CV026 lawn for 24 h at 28°C; **(B)** quantification of AHLs production abilities, ***p < 0.001.

**FIGURE 8:**
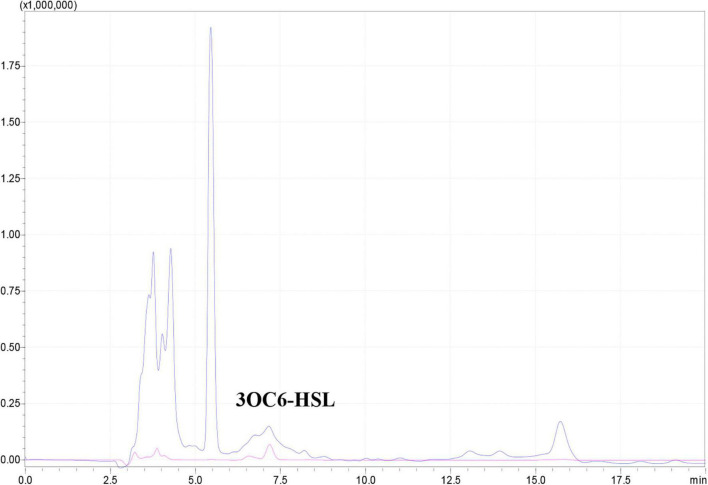
3OC6-HSL produced by *Dickeya zeae* strain WH1 measured by HPLC. Blue indicates 3OC6-HSL present in the supernatant from *D. zeae* strain WH1; pink indicates commercial 3OC6-HSL standard.

The *exp*I/*exp*R quorum sensing system cluster was highly conserved in the five genomes. Therefore, AHL produced by WH1 was qualified with CV026. The results showed that WH1 produced more AHLs than QS model strain *P. aeruginosa* PAO1 when incubated at the same conditions (Figure 7). Additionally, the results of HPLC demonstrated that the AHL produced by WH1 was 3OC6-HSL (Figure 8).

### Pathogenicity assays

**FIGURE 9:**
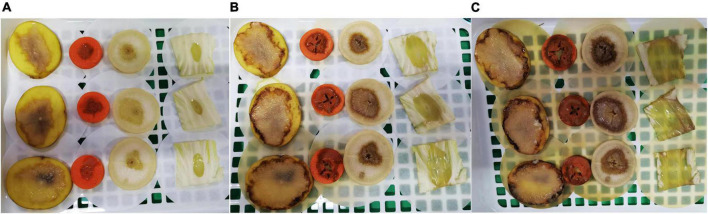
Pathogenicity of *Dickeya zeae* strain WH1 on potato tubers, carrot, radish, and Chinese cabbage. **(A)** Infected for 24 h; **(B)** infected for 48 h; **(C)** infected for 72 h.

**FIGURE 10:**
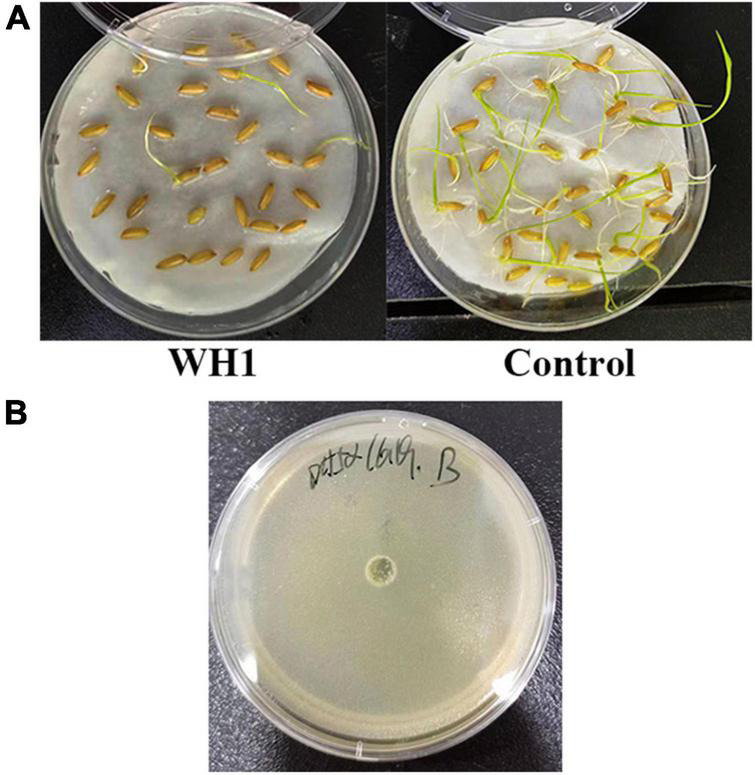
Phytotoxins production by *Dickeya zeae* strain WH1. **(A)** Inhibitory activity of toxins from *D. zeae* strain WH1 against rice seeds germination; **(B)** the WH1 culture did not inhibit the growth of *Escherichia coli* DH5α.

The results of pathogenicity tests on potato tubers, carrots, radishes, and Chinese cabbage confirmed that the WH1 strain infected all plants with serious maceration symptoms. When infected for 72 h, almost plant tissues were macerated (Figure 9). We also tested the inhibitory activity of WH1 against rice seed germination. The results indicated that WH1 had a strong inhibitory effect on rice seed germination with an inhibitory rate being about 87% (Figure 10A). Zeamine detection assay demonstrated that WH1 did not inhibit the growth of *E. coli* DH5α (Figure 10B). Therefore, the virulence factors inhibiting rice seed germination were not zeamines.

## Discussion

Soft rot *Pectobacteriacaea* (SRP) includes the genera *Pectobacterium* and *Dickeya* and are widespread globally, which cause blackleg disease in potatoes and soft rot disease in a wide range of hosts, including vegetables, fruits, ornamental plants, and the most important crops, rice, and maize. Surprisingly, there is no report on *Pectobacterium* spp. isolated from rice and maize. In contrast, *Dickeya* spp. was isolated from a wide range of hosts compared to *Pectobacterium* spp. (Toth et al., 2021). Although there are some differences in isolated hosts, SRP shares many virulence factors, including PCWDE, secretion systems, motility, and plant responses to SRP infections (Davidsson et al., 2013; Reverchon et al., 2016). In *Dickeya* genus, *D. zeae* strains and their genomes were used for references to study virulence factors and pathogenicity of the genus (Zhang et al., 2014, 2022; Zhou et al., 2015; Lv et al., 2022). However, there is a relatively small amount of research on the above diseases in rice (Van Gijsegem et al., 2021).

Research to date indicates that *D. zeae* strains can infect a wide range of host plants, including 24 dicotyledonous plants and 23 monocotyledonous plants (Hu et al., 2022). Therefore, both crop rotation and interplants might be impacted once infected by this kind of strain. Currently, most *Dickeya* strains were isolated from diseased plants. In this study, although WH1 was initially isolated from the healthy rice root surface, the pure culture has serious pathogenicity on rice and other plants. Additionally, a potential biocontrol strain, *P. polymyxa*, also isolated from the healthy rice root surface, can significantly inhibit the growth of WH1. Importantly, *D. zeae* are often present in latent infections on many host crops and they can persist over winter in contaminated plant residues and soil. When the optimal conditions of temperature, humidity, and other factors occur, latency is broken and bacteria proliferate and cause decay (Bez et al., 2021). Therefore, the rice and other plants are grown in the field infected by WH1 face the risk of soft rot disease once microbial balance leads to a high abundance of the *D. zeae* strain.

Based on our analysis, we conclude that the novel strain WH1 is diverse and phylogenetically close to *D. zeae* strain EC2 isolated from rice in Guangdong, China. Given that the 16S rRNA gene sequence is insufficient to assign the taxonomy of the bacterium, the joint phylogenetic tree based on MLSA analysis was considered. We found that WH1, EC1, and EC2 were grouped into a clade, even though WH1 and EC1, and EC2 were isolated from different places (Eastern China and Southern China). Meanwhile, the results of ANI and DDH analyses also confirmed the MLSA analysis. Interestingly, EC1 harbors a *zms* gene cluster involving zeamines in biosynthesis, which can inhibit the growth of *E. coli* DH5α and rice seed germination (Hu et al., 2018), was absent in WH1 and EC2 strains isolated from rice and MS1 and MS2 isolated from banana. However, WH1 produced phytotoxin(s) with high inhibitory activity on rice seed germination and no effect on the growth of *E. coli* DH5α, which further revealed that WH1 produces other toxins instead of zeamines. Additionally, we found WH1 can inhibit the growth of *Candida albicans*, while *P. aeruginosa* PAO1 can inhibit the growth of WH1, which was different from EC1 and MS2 (Hu et al., 2018). Therefore, the toxins produced by WH1 would be different from other *D. zeae* strains, especially different from EC1. The toxins produced by WH1 require further exploration in the future.

Plant cell wall degrading enzymes produced by the *Dickeya* genus are considered essential virulence factors for host plants and disease development (Zhang et al., 2020). Our genomic comparative analyses demonstrated that the main encoding genes associated PCWDEs were existed and conserved in WH1 and other *D. zeae* strains. Besides, the pathogenesis of *Dickeya* spp. involved in several complex secretion systems (T1SS–T6SS) to translocate a wide range of extracellular enzymes and effector proteins from the periplasm across the outer membrane. The Prts, which are crucial for virulence, are secreted by the T1SS known as the *prt*DEF operon. The T1SS cluster, mainly transporting metalloproteinase, was present in all analyzed strains. Previous studies have revealed that *Dickeya* species mainly used the T2SS to translocate extracellular proteins such as pectinases and cellulases. Similar results were obtained in our analyses. Additionally, Hu et al found that the deletion of T3SS substantially diminished MS2’s pathogenicity on both dicots and monocots (Hu et al., 2022). Meanwhile, they suggested that the loss of T3SS and reduced PCWDE activity together might have contributed to the host specificity and virulence of *D. zeae* JZL7, one of the monocot-specific strains isolated from *Clivia miniata*. The effect of T3SS on WH1’s pathogenicity will therefore require further investigation in the future. T6SS was recognized as a distinct class of bacterial protein secretion system and identified as a virulence factor in Gram-negative bacteria (Chen et al., 2015). However, the biological function of T6SS in the *Dickeya* genus has not yet been determined. In this study, a gene cluster encoding T6SS was present in all analyzed genomes.

Among Gram-negative bacteria, EPS and CPS are often essential virulence determinants in plant pathogens. EPS is a main component of the bacterial biofilm matrix and is responsible for adhesion to plant surfaces (Chen et al., 2015; Zhang et al., 2020). Strains EC1 and EC2 isolated from rice produced either EPS or CPS, while banana isolates MS1 and MS2 generally produced significantly more EPS than strains from rice and exhibited different biofilm-associated phenotypes (Hu et al., 2018). A previous study demonstrated that MS1 could form a thick biofilm, while EC1 biofilm appeared in clumps and EC2 biofilm was thinner (Zhang et al., 2020). WH1 had the entire EPS and CPS gene clusters, and our study found that the strain easily formed thick biofilm *in vitro*. Therefore, the relationship between the ability of WH1 to form biofilm and its pathogenicity will be required investigation in the future. Flagellar is used for both swimming and swarming motility. Jahn et al. (2008) have proven that the deletion of the *fli*A gene encoding a sigma factor obstructed bacterial motility, and limited Pel production and bacterial attachment to plant tissues in *D. dadantii* 3937. Moreover, the type IV pilus was responsible for motility twitching in *P. aeruginosa* and *Dickeya aquatic* (Duprey et al., 2019). Therefore, flagellar biosynthesis and chemotaxis proteins and the type IV pilus play important roles in plant bacterial pathogenicity. We found these clusters present in all analyzed *D. zeae* strains.

*Dickeya* species have at least two QS systems, *exp*I/*exp*R, and *vfm* systems. A previous study demonstrated that deletion of *exp*I in *D. zeae* EC1 abolished AHL production, increased swimming and swarming motility, disabled biofilm formation, attenuated virulence of pathogen on potato tubers, and reduced the inhibitory activity on rice seed germination through decreasing the expression of *zms* genes (Hussain et al., 2008). The inactivation of *exp*I in *D. zeae* MS2 affected motility and cell clumping, while it did not affect infections of banana seedlings (Feng et al., 2019). Therefore, the effects of the *exp*I/*exp*R system on the virulence and pathogenicity of *D. zeae* strains is complex. The genome of WH1 also contains *exp*I/*exp*R clusters with an identity of 99% with other *D. zeae* strains. Meanwhile, HPLC demonstrated the AHL produced by WH1 was 3OC6-HSL. However, there are different virulence determinants between WH1 and EC1, such as the zeamine biosynthesis gene cluster that is present in EC1 but absent in WH1. Therefore, the effect of *exp*I/*exp*R system on virulence factors production of WH1 remains to further investigate in the future.

Vfm-QS signal is uniquely present in *Dickeya* strains, participating in and dominating the macerating soft rot process of the pathogen in plant tissues. Previous studies demonstrated that the *vfm* QS system participated in the regulation of PCWDEs production and virulence in the *Dickeya* genus (Zhang et al., 2014). However, the chemical structure of the Vfm signal has not been characterized yet. In this study, the entire *vfm* gene cluster was found in the WH1 genome, but its effect on virulence has not yet been investigated through experimental evidence.

## Conclusion

This study presents a high-quality complete genome sequence of a novel *D. zeae* strain WH1 isolated from rice root. Phylogenetic tree analysis and genome-genome nucleotide comparison indicated that WH1 was placed at the same branch with EC2 isolated from rice. The genome sequence of WH1 was annotated and compared with the representative genome sequences of other *D. zeae* strains, focusing on virulence determinants and potential regulatory mechanisms. Several groups of virulence genes, such as those encoding for PCWDEs, T1SS gene cluster, T2SS gene cluster, T6SS gene cluster, flagellar and chemotaxis gene cluster, and quorum sensing gene cluster were highly or fully conserved in all five genomes isolated from different hosts. Interestingly, the T3SS gene cluster was absent in strain EC2, T4SS gene cluster was absent in WH1, EC2, and MS2. Genes inserted between *vgr*G and *tss*B within the T6SS gene cluster were different in all analyzed genomes. Both EPS and CPS gene clusters were present in WH1, MS1, and MS2, while either EPS or CPS was present in EC1 or EC2. Gene cluster encoding zeamines biosynthesis was only present in EC1. Additionally, related phenotypic and pathogenicity analyses were performed for strain WH1. The strain can produce pectate lyases, cellulases, and proteases, easily formed thick biofilm *in vitro*, and had strong AHL quorum sensing activity. Besides, WH1 can infect plants with serious maceration symptoms and strongly inhibit rice seed germination through other toxins instead of zeamines.

## Data availability statement

The datasets presented in this study can be found in online repositories. The names of the repository/repositories and accession number(s) can be found below: https://www.ncbi.nlm.nih.gov/PRJNA858112, CP101400.

## Author contributions

X-JT, G-PZ, and YS conceived and designed the experiments and revised the manuscript. Z-WZ, J-JX, WW, FH, BJ, and XW performed the experiments. X-JT, XG, and LS analyzed the data. X-JT wrote the manuscript. All authors contributed to the article and approved the submitted version.

## Footnotes

1
https://ggdc.dsmz.de/home.php
