# Supplementary material for: Genomic and phenotypic biology of a novel Dickeya zeae WH1 isolated from rice in China: Insights into pathogenicity and virulence factors
Source: Front Microbiol. 2022 Oct 28;13:997486. doi: 10.3389/fmicb.2022.997486 (PMC9650423; doi:10.3389/fmicb.2022.997486)
Supplement: Supplementary file 1 [file Data_Sheet_1.PDF]

A

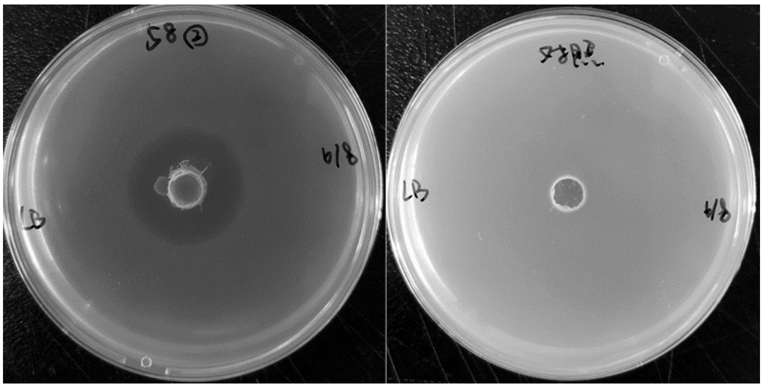

B

select all *100 sequences selected* GenBank Graphics Distance tree of results MSA Viewer

Description	Scientific Name	Max Score	Total Score	Query Cover	E value	Per. Ident	Acc. Len	Accession
Paenibacillus polymyxa strain 28 16S ribosomal RNA gene, partial sequence	Paenibacillus polymyxa	2612	2612	98%	0.0	99.38%	1460	HQ259963.1
Paenibacillus sp. WS25 16S ribosomal RNA gene, partial sequence	Paenibacillus sp. WS25	2610	2610	98%	0.0	99.44%	1457	JN899575.1
Paenibacillus sp. strain BYT-3-2 16S ribosomal RNA gene, partial sequence	Paenibacillus sp.	2606	2606	98%	0.0	99.38%	1460	OL654403.1
Paenibacillus polymyxa strain DSM 36 16S ribosomal RNA, partial sequence	Paenibacillus polymyxa	2604	2604	98%	0.0	99.17%	1547	NR_117733.2
Paenibacillus polymyxa strain C12 chromosome, complete genome	Paenibacillus polymyxa	2604	35652	98%	0.0	99.17%	5793823	CP023711.1
Paenibacillus sp. strain JK1 16S ribosomal RNA gene, partial sequence	Paenibacillus sp.	2604	2604	98%	0.0	99.17%	1547	OM432155.1
Paenibacillus polymyxa partial 16S rRNA gene, type strain DSM 36T, clone 14	Paenibacillus polymyxa	2604	2604	98%	0.0	99.17%	1604	HG324077.1
Paenibacillus polymyxa strain HY96-2 chromosome, complete genome	Paenibacillus polymyxa	2599	35864	98%	0.0	99.10%	5745779	CP025957.1
Paenibacillus polymyxa strain YS18-2 chromosome, complete genome	Paenibacillus polymyxa	2599	36013	98%	0.0	99.10%	5840603	CP085682.1
Paenibacillus polymyxa strain O 2.11 chromosome, complete genome	Paenibacillus polymyxa	2599	33298	98%	0.0	99.10%	5495678	CP097773.1
Paenibacillus polymyxa strain R 6.14 chromosome, complete genome	Paenibacillus polymyxa	2599	35886	98%	0.0	99.10%	5745819	CP097766.1
Paenibacillus polymyxa strain O 1.27 chromosome, complete genome	Paenibacillus polymyxa	2599	33298	98%	0.0	99.10%	5493315	CP097775.1
Paenibacillus polymyxa strain ZJ-9 chromosome, complete genome	Paenibacillus polymyxa	2599	35842	98%	0.0	99.10%	5755887	CP092779.1
Paenibacillus polymyxa SQR-21, complete genome	Paenibacillus polymyxa SQR-21	2599	32934	98%	0.0	99.10%	5828436	CP006872.1
Paenibacillus jamilae partial 16S rRNA gene, strain KCTC 13919, clone 12	Paenibacillus jamilae	2599	2599	98%	0.0	99.10%	1476	HE981804.1
Paenibacillus sp. YP21 16S ribosomal RNA gene, partial sequence	Paenibacillus sp. YP21	2595	2595	98%	0.0	99.24%	1461	KF719298.1
Paenibacillus sp. YP8 16S ribosomal RNA gene, partial sequence	Paenibacillus sp. YP8	2595	2595	98%	0.0	99.17%	1495	KF719293.1
Paenibacillus polymyxa strain Pp-HK18-5 16S ribosomal RNA gene, partial sequence	Paenibacillus polymyxa	2595	2595	97%	0.0	99.58%	1432	MF490457.1
Paenibacillus sp. WP18 16S ribosomal RNA gene, partial sequence	Paenibacillus sp. WP18	2593	2593	98%	0.0	99.24%	1494	KF719303.1
Paenibacillus polymyxa strain O 2.11 plasmid pPpO211, complete sequence	Paenibacillus polymyxa	2593	2593	98%	0.0	99.03%	248380	CP097774.1
Paenibacillus polymyxa strain O 1.27 plasmid pPpO127_1, complete sequence	Paenibacillus polymyxa	2593	2593	98%	0.0	99.03%	265069	CP097776.1
Paenibacillus polymyxa strain R 3.13 chromosome, complete genome	Paenibacillus polymyxa	2593	35735	98%	0.0	99.03%	5730787	CP097771.1

Supplementary Figure 1|Inhibitory effect of strain 58 on the growth of *D. zeae* strain WH1 (A) and its 16S rRNA gene sequence alignment result (B). Inhibitory activity of 20 µL of strain 58 overnight culture (left) against WH1 growth and 20 µL of LB as negative control (right).

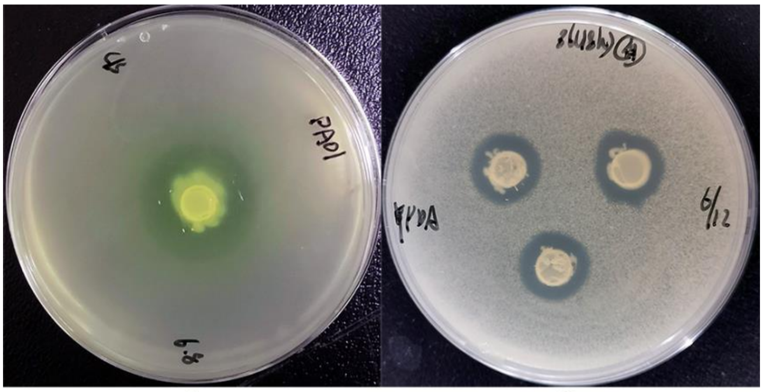

A B

Supplementary Figure 2| Inhibitory effect of *Pseudomonas aeruginosa* PAO1 on the growth of *D. zeae* strain WH1 (A) and inhibitory effect of WH1 on the growth of *Candida albicans* (B).
